# PERSPECTIVES ON SETTING GOALS OF CARE AMONG HOSPICE PATIENTS WITH HEART FAILURE, THEIR FAMILIES, AND HEALTHCARE TEAM

**DOI:** 10.1093/geroni/igz038.2914

**Published:** 2019-11-08

**Authors:** Dawon Baik, David Russell, Lizeyka Jordan, Frances Dooley, Ruth Masterson Creber

**Affiliations:** 1 Columbia University, New York, New York, United States; 2 Appalachian State University, Visiting Nurse Service of new York, Boone, North Carolina, United States; 3 Visiting Nurse Service of New York, New York, New York, United States; 4 Weill Cornell Medicine, New York, New York, United States

## Abstract

Older adults with heart failure (HF) face many end-of-life care issues. Shared decision making (SDM) in hospice is an important process that allows HF patients and their family caregivers to discuss their preferences on goals of care (GOC) with their healthcare team. Yet, little research has explored how the values and preferences of HF patients and their family are integrated into their care plans through SDM process. This presentation examines facilitators and barriers to setting GOC among hospice HF patients. Qualitative interviews were conducted with HF patients/family caregivers (n=7) and providers (n=32) at a large not-for-profit hospice agency. Several facilitators emerged: building trust, active listening, helping patients and family caregivers understand hospice and prognosis. Barriers included acceptance, family conflict, language discordance between patients and providers and lack of communication about care transition. Findings confirmed the need for individually-tailored goal-setting approaches to navigating the end-of-life trajectory among HF patients.

